# Oxford University

**Published:** 1914-09-05

**Authors:** 


### OXFORD UNIVERSITY COURSE.

At Oxford University two degrees in medicine can be obtained, the B.M. and the D.M.; similarly in surgery there are two degrees, the B.Ch. and the M.Ch. No candidate is eligible for any of these degrees unless he is a graduate in Arts, that is to say, either an M.A. or a B.A.; in which branch of the Arts that degree has been obtained is a matter of little moment. The usual course of the medical student who has passed Responsions is to work at the Preliminary Science examinations in physics, chemistry, and zoology and botany, which are likely to take him two years or thereabouts, and then to spend a further two years in study for the Final Honour School of physiology, in which he takes his degree of B.A. He next has to pass examinations in organic chemistry in relation to medicine and in human anatomy, after which he prepares himself for his second professional examination, which comprises the subjects of materia medica and pharmacology, pathology, forensic medicine and hygiene, and the Final subjects of medicine, surgery, and midwifery. When the examinations in these subjects have been passed, the candidate may supplicate for and obtain the degree of B.M., B.Ch.

The degree of D.M. is granted to Bachelors of Medicine of the University who have entered their thirty-ninth term —it should be remembered that there are four terms in the official year—who have presented a dissertation on some medical subject that is approved by the Professors of the Faculty and Examiners for the degree of Bachelor of Medicine for the time being whose subjects are dealt with in it. The degree of M.Ch. is granted to Bachelors of Surgery of the University who have entered their twenty-seventh term and satisfied some other requirements; the examination for this degree is held annually in June.

